# The Perioperative Use of Levosimendan as a Means of Optimizing the Surgical Outcome in Patients with Severe Heart Insufficiency Undergoing Cardiac Surgery

**DOI:** 10.3390/jcdd10080332

**Published:** 2023-08-03

**Authors:** Vasileios Leivaditis, Konstantinos Grapatsas, Anastasia Papaporfyriou, Michail Galanis, Efstratios Koletsis, Nikolaos Charokopos, Erich Haussmann, Vladislav Kaplunov, Athanasios Papatriantafyllou, Manfred Dahm

**Affiliations:** 1 Department of Cardiothoracic and Vascular Surgery, Westpfalz-Klinikum, 67655 Kaiserslautern, Germany; vnleivaditis@gmail.com (V.L.); ehaussmann@westpfalz-klinikum.de (E.H.); vkaplunov@westpfalz-klinikum.de (V.K.); mdahm@westpfalz-klinikum.de (M.D.); 2 Department of Thoracic Surgery and Thoracic Endoscopy, Ruhrlandklinik, West German Lung Center, University Hospital Essen, University Duisburg-Essen, 45239 Essen, Germany; 3 Department of Pulmonology, Internal Medicine II, Vienna University Hospital, 1090 Vienna, Austria; anastasia.papaporfyriou@meduniwien.ac.at; 4 Department of Thoracic Surgery, Inselspital—Bern University Hospital, University of Bern, 3012 Bern, Switzerland; michail.galanis@insel.ch; 5 Department of Cardiothoracic Surgery, University Hospital of Patras, 26504 Patras, Greece; ekoletsis@hotmail.com (E.K.); nc.7@hotmail.com (N.C.); 6 Department of General Surgery, General Hospital of Patras “Agios Andreas”, 26332 Patras, Greece; thanospap9@yahoo.gr

**Keywords:** levosimendan, heart failure, cardiac surgery, myocardial injury, angina pectoris

## Abstract

Background: Postoperative myocardial dysfunction following cardiac surgery is a relatively common occurrence. Levosimendan, a calcium sensitizer and inotropic drug, has shown potential in improving outcomes for patients with low preoperative ejection fraction (EF) and myocardial dysfunction after cardiac surgery. This study aims to evaluate the efficacy of levosimendan in optimizing the surgical outcome for such patients. Methods: A retrospective analysis was conducted on 314 patients with preoperative severe heart failure who underwent cardiac surgery. Among them, 184 patients received perioperative adjunctive therapy with levosimendan, while a comparable group of 130 patients received conventional treatment. Results: The use of levosimendan demonstrated several advantages in postoperative outcomes. It significantly improved short- and long-term survival rates after cardiac surgery, enhanced hemodynamic stability, reduced the requirement for inotropic support, and facilitated faster weaning from ventilator support. Patients who received levosimendan reported reduced angina and dyspnea symptoms, as well as fewer postoperative arrhythmias. Furthermore, levosimendan helped minimize myocardial injury inevitable after cardiac surgery. The levosimendan group also exhibited a notable reduction in hospital readmissions. Conclusions: This study provides evidence of several benefits associated with the perioperative use of levosimendan. However, further prospective randomized studies are warranted to standardize and comprehensively document the other perioperative therapies, in order to validate these findings and establish stronger conclusions.

## 1. Introduction

Postoperative myocardial dysfunction is a common occurrence after cardiac surgery, affecting both patients with normal preoperative ventricular function and those with pre-existing heart failure. The severity of this dysfunction is influenced by various factors, including patient age, type of procedure, and existing comorbidities. In patients with pre-existing heart failure, postoperative myocardial dysfunction can lead to complications such as delayed recovery, organ failure, prolonged stays in the intensive care unit (ICU) and hospital, increased morbidity and mortality, and higher healthcare costs. Significant myocardial dysfunction can compromise tissue perfusion, necessitating the use of inotropic agents to provide hemodynamic support until endogenous myocardial function is restored [1,2,3].

Traditional treatment strategies primarily involve the use of catecholamines, particularly β-adrenergic agents, which are considered first-line therapy due to their positive effects on myocardial contractility and cardiac output. However, the use of these sympathomimetic drugs is associated with several side-effects, including tachycardia, arrhythmias, adverse effects on ventricular load conditions, and potential disruptions in myocardial oxygen supply and consumption. Moreover, in patients with chronic heart failure, downregulation of β-adrenergic receptors can attenuate the response to exogenously administered catecholamines [1,2].

Levosimendan, an inotropic drug belonging to the class of calcium sensitizers, offers an alternative approach. It enhances myofilament calcium sensitivity during cardiac systole by binding to troponin C in a calcium-dependent manner, thereby stabilizing the calcium-induced conformational change of tropomyosin. This mechanism augments actin-myosin cross-bridging without increasing intracellular calcium concentrations. Additionally, levosimendan binds to and activates ATP-dependent potassium (K_ATP_) channels, directly influencing vascular smooth muscle cells and causing vasodilation. The beneficial effects of levosimendan have been described in various studies involving internal medicine and surgical patients, although controversies remain [2,3].

The aim of this study was to investigate whether the advantages of levosimendan can be extended to improve the outcomes of patients with low preoperative ejection fraction (EF) and myocardial dysfunction following cardiac surgery. By evaluating the potential benefits of levosimendan in this specific patient population, we aim to contribute to the existing knowledge and provide insights into optimizing surgical outcomes in individuals with severe heart insufficiency.

## 2. Materials and Methods

### 2.1. Study Design and Patient Data Collection

The present study constitutes a retrospective analysis utilizing data collected from a cohort of 314 consecutive patients who underwent cardiac surgery between the years 2008 and 2017 at the Department of Cardiothoracic and Vascular Surgery in Westpfalz-Klinikum Hospital, Kaiserslautern. Specifically, the study focuses on patients with pre-existing heart failure who underwent cardiac surgery. Among the selected cases, 184 patients received a preoperative and perioperative additive therapy involving the administration of the calcium sensitizer, levosimendan. A control group consisting of 130 patients with similar medical history was included, and these individuals received conventional treatment. To ensure the inclusion of patients from a comparable time frame, the individuals in the historical control group were recorded in a manner that acknowledges the inherent limitations of retrospective studies. Although the patient allocation was not randomized, it was performed in a random and comparable manner within the context of this retrospective analysis.

All patients included in the study received postoperative care in the ICU and were subsequently transferred to the intermediate care unit before being transferred to the general ward. Patients who were administered levosimendan were provided comprehensive information regarding its administration, effects, and potential side-effects. Informed consent was obtained from these patients after they were adequately informed. The collection of data was facilitated through the central digital archive of Westpfalz-Klinikum hospital in Kaiserslautern. The documentation of primary data commenced upon hospital admission and continued until the patient’s discharge, transfer to another hospital, or death. Laboratory parameters were extracted from the standard software program utilized at Westpfalz-Klinikum hospital. Follow-up data were obtained either from our archive or through direct communication with the patients, their relatives, or their primary care physician. All patients received treatment according to the established guidelines of the European Society of Cardiology (ESC) and European Association of Cardiothoracic Surgery (EACTS), with individualized exceptions made in exceptional cases.

### 2.2. Inclusion Criteria

The study included adult patients who underwent cardiac surgery with the assistance of extracorporeal circulation and were preoperatively diagnosed with heart failure. All patients exhibited advanced heart failure, classified as the New York Heart Association (NYHA) Class III–IV and Stage D according to the ACC/AHA (American College of Cardiology/American Heart Association) 2005 classification system. This classification was based on clinical signs of decompensation, along with a preoperative left-ventricular ejection fraction (LVEF) of ≤30% as determined by transthoracic echocardiography (TTE), transesophageal echocardiography (TEE), or magnetic resonance imaging (MRI). These patients demonstrated indications for therapy with positive inotropic agents.

### 2.3. Exclusion Criteria

Patients with a preoperative left-ventricular ejection fraction (LVEF) higher than 30% as determined by TTE, TEE, or MRI were not included in the study. Furthermore, patients who were managed conservatively with medication or nonsurgical interventions such as percutaneous coronary intervention (PCI) or transcatheter aortic valve implantation (TAVI), among others, were also excluded. Additionally, patients with acute endocarditis, severe acute or chronic liver disease, systemic inflammatory syndrome (SIRS), or sepsis within the 2 weeks preceding surgery were excluded. Patients who could not tolerate levosimendan administration due to excessive vasodilatory effects, as mentioned previously, were also excluded. Moreover, individuals under the age of 18, those who did not provide consent for the administration of levosimendan, or those participating in another study were also excluded from this study.

### 2.4. Application and Dosis of Levosimendan

In our study, all patients from both groups received the levosimendan treatment under the exact same protocol. The active substance used in this study was levosimendan, which was administered in the form of Simdax^®^ by Orion Pharma (Orion Pharma GmbH, Hamburg, Germany). Each milliliter of the concentrate contained 2.5 mg of levosimendan. The infusion solution was prepared by mixing 12.5 mg of the active ingredient with 50 mL of 0.9% sodium chloride (NaCl) solution, resulting in a concentration of 0.25 mg/mL. The infusion was administered over a period of 24 h, and a bolus dose was not utilized in this patient cohort. The administration of levosimendan was consistently performed preoperatively, and it commenced 24 h before the operation. The drug was administered to the patients while they were in the intensive care unit (ICU) and was discontinued when the patient was transferred to the operating theater for the cardiac surgery procedure. All patients received levosimendan at a dose of 0.1 μg/kg/min, adjusted according to the hemodynamic status of the patient. The administration of levosimendan was carried out through both central venous and peripheral venous accesses. Patients with mild to moderate impairment of renal or hepatic function were closely monitored during the administration process. The infusion took place in ICU, where vital parameters were continuously monitored. Blood pressure monitoring was achieved invasively through radial artery catheterization in all patients. Attention was given to assessing the intravascular volume status of these patients and providing appropriate infusion therapy. In the presence of vasodilatory effects, patients were treated with low-dose norepinephrine. However, if persistent hypotension occurred despite additional support with intravenous fluids and norepinephrine, the levosimendan administration was discontinued. These patients were subsequently excluded from the study.

### 2.5. Ethics

The study was conducted following the principles outlined in the Declaration of Helsinki and received approval from the Ethics Commission of the State Medical Association (Landesärztekammer) of Rheinland-Palatinate. A favorable ethical decision was obtained on 16 June 2015, considering the regulations specified in the State Hospital Act (Landeskrankenhausgesetz §36 and §37) and the guidelines of good clinical practice for trials involving medical products in the European community (ICH-GCP).

### 2.6. Statistical Analysis

Categorical variables were summarized using frequencies and percentages, while continuous variables were described using the median, interquartile range (IQR), and mean (if the distribution was approximately normal). The distribution of continuous variables was visualized using histograms, while bar charts were used for categorical variables.

Appropriate statistical tests were applied on the basis of the nature of the variables to determine if there were statistically significant differences between the two groups. The chi-squared test was employed to assess the association of categorical variables, while the Kruskal–Wallis test was used to evaluate the association between an ordinal variable and a categorical variable. The distribution of continuous variables in each group was assessed through histograms or the Shapiro–Wilk test. Depending on the distribution, either the *t*-test or Mann–Whitney test was used for comparisons. The significance level for all statistical tests was set at *p* < 0.05.

To estimate the probability of survival after heart surgery in the two groups, the Kaplan–Meier method was utilized. Survival estimates at different timepoints, along with their 95% confidence intervals, are presented. The log-rank test was performed to assess whether there was a significant difference in survival functions between the two groups. Statistical analysis was conducted using STATA 13.

### 2.7. Measured Parameters

Survival was the major endpoint of this study. Short-term (10 days and 30 days) and long-term (6 months, 1 year, 2 years, and 3 years) survival rates were recorded. The evaluation of postoperative left ventricular ejection fraction (EF) and the calculation of the difference (ΔEF = EF_postop_ − EF_preop_) between preoperative and postoperative EF were conducted for all patients.

Blood chemistry laboratory parameters, including hemoglobin, troponin I, creatine kinase (CK), and myocardial creatine kinase (CK-MB), were utilized to assess organ function and the extent of myocardial damage. These values were collected before surgery, upon the patient’s arrival in the ICU, and during the first 5 days after surgery. Arterial blood gas analysis was performed daily in the ICU to monitor respiratory and metabolic status.

Additional parameters, such as invasive ventilation time, duration of weaning from the ventilator, perioperative requirement for inotropic support, use of mechanical support with an intra-aortic balloon pump (IABP), and frequency of postoperative arrhythmias, were recorded and compared between the two groups. The duration of the total hospital stay and ICU stay was also compared between the two groups.

During follow-up, patients were assessed for angina and dyspneic symptoms. Symptom severity was classified using grading scales such as the Canadian Cardiovascular Society (CCS) and New York Heart Association (NYHA). The number of hospital readmissions due to cardiac causes within 3 years after surgery was documented by reviewing hospital archives and through direct communication with patients or their general practitioners during follow-up.

## 3. Results

### 3.1. Demographics

Although this study had a retrospective design, the patient groups demonstrated homogeneity in terms of general demographic data. Among the participants, 184 patients received levosimendan treatment, while the control group consisted of 130 patients who received conventional treatment and exhibited similar medical history and clinical characteristics. Table 1 and Table 2 present the baseline demographic, biological, and clinical characteristics of the study population.

The distribution of gender was comparable between the control and levosimendan groups, with males constituting the majority in both groups, accounting for 81.54% and 78.80%, respectively (*p* = 0.551). There was no statistically significant difference observed in the median age of operation between the control group (71 years) and the levosimendan group (69 years) (*p* = 0.703). Furthermore, no significant differences were found in the distributions of weight (*p* = 0.087), height (*p* = 0.874), body mass index (BMI) (*p* = 0.072), and body surface area (BSA) (*p* = 0.096) between the two groups.

### 3.2. Clinical Characteristics at Baseline

The collected data regarding preoperative clinical characteristics of patients who took part in this study are presented in Table 2.

The baseline Euroscore 1 values were significantly higher in the levosimendan group (22.8%) compared to the control group (15.72%) (*p* < 0.001). Similarly, the baseline Euroscore 2 values were significantly higher in the levosimendan group (8.82%) compared to the control group (5.43%) (*p* < 0.001). However, odds ratio (OR) and 95% confidence interval (CI) values were not significantly different between the two groups. These findings indicate that patients in the levosimendan group had tendency for a higher perioperative risk than those in the control group, which should be taken into account when comparing survival rates between the two groups. Additionally, a significant difference was observed in the distribution of American Society of Anesthesiologists (ASA) physical status (*p* = 0.048). Although most patients in both groups were classified as ASA class 4, a relatively higher proportion of patients in the levosimendan group were classified as ASA 4 and 5, indicating a higher preoperative risk in this group. ORa and 95% CI values also showed here no significant differences.

Ejection fraction (EF) was a critical factor and a major inclusion criterion in this study. Preoperative baseline values of EF were evaluated to assess the improvement after treatment. The control group had higher values of EF compared to the levosimendan group (30% and 20% respectively, *p* < 0.001), indicating a higher grade of heart failure in the levosimendan group. However, there was no significant difference in the occurrence of acute myocardial infarctions between the two groups (27.69% in the control group and 25.54% in the levosimendan group, *p* = 0.671).

Regarding dyspnea staging according to NYHA classification, a higher percentage of patients in the control group experienced Stage 3 preoperatively (50.76%) compared to the drug group (31.13%) (*p* = 0.032). Conversely, NYHA Stage 4 was more prominent in the levosimendan group (56.52% vs. 43.85% in the control group, *p* = 0.032). The staging of angina pectoris showed a similar pattern between the two groups, with 46.15% of the control group and 33% of the levosimendan group experiencing symptoms during ordinary physical activity (Class 3), while 33.07% of the control group and 44.57% of the levosimendan group reported angina pectoris at rest (Class 4) (*p* = 0.124).

Not all patients included in this study underwent elective regular surgery, as a significant number of them required urgent operations. The majority of cases underwent elective/regular surgery with no significant difference between the control group (73.85%) and the levosimendan group (72.28%) (*p* = 0.759). In terms of multiple surgical procedures, 26.92% of the control group and 35.85% of the levosimendan group required more than one procedure (e.g., bypass grafting, aortic or mitral valve replacement or repair, and surgery on the aorta or aortic root), but this difference was not statistically significant (*p* = 0.095).

Arrhythmias and pulmonary hypertension were more prevalent in the levosimendan group compared to the control group (30.43% vs. 19.23%, *p* = 0.030 and 30.43% vs. 8.46%, *p* < 0.001, respectively). The following risk factors, including arterial hypertension (*p* = 0.321), diabetes mellitus (*p* = 0.353), hyperlipidemia (*p* = 0.175), and COPD (*p* = 0.118), did not show significant differences between the two groups, indicating a similar comorbidity profile. However, patients in the levosimendan group tended to have a higher prevalence of renal failure, extracardial arteriopathy, and cerebrovascular accident compared to patients in the control group (25.54% vs. 13.85%, *p* = 0.012; 31.52% vs. 16.28%, *p* = 0.003; and 13.59% vs. 4.62%, *p* = 0.009, respectively). OR and 95% CI values did not, however, show any significant difference for arrhythmias, extracardiac arteriopathy, and renal failure between the two groups.

### 3.3. Overall Survival

Survival rates were assessed and compared between both groups at specific timepoints, including at 10 days, 30 days, 6 months, and 1, 2, and 3 years post surgery, as presented in Table 3.

**Figure 1:**
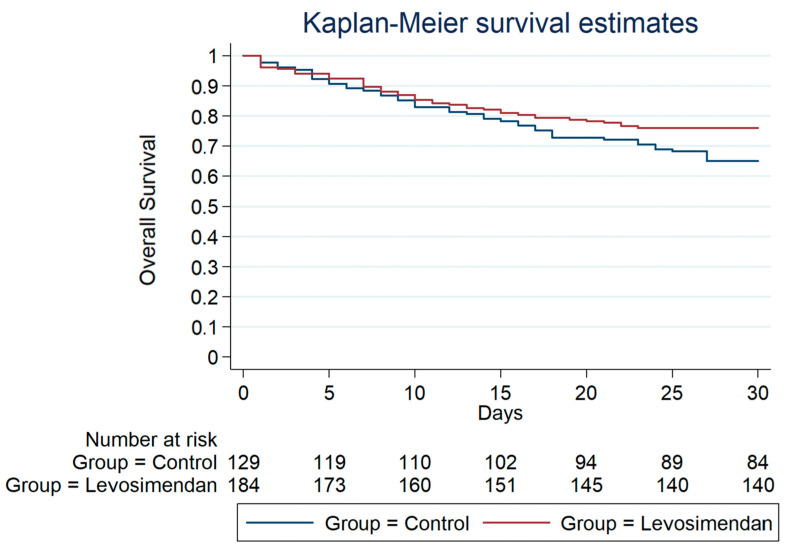
Overall survival from the date of operation within 30 days after surgery.

**Figure 2:**
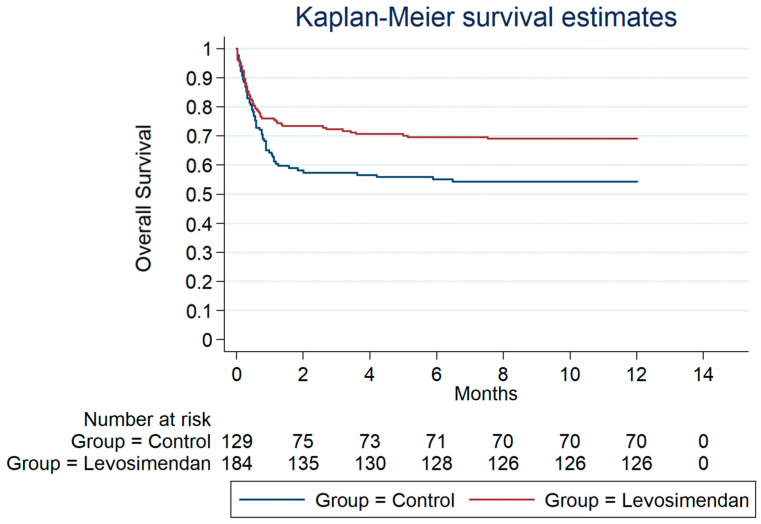
Overall survival from the date of operation within 1 year after surgery.

**Figure 3:**
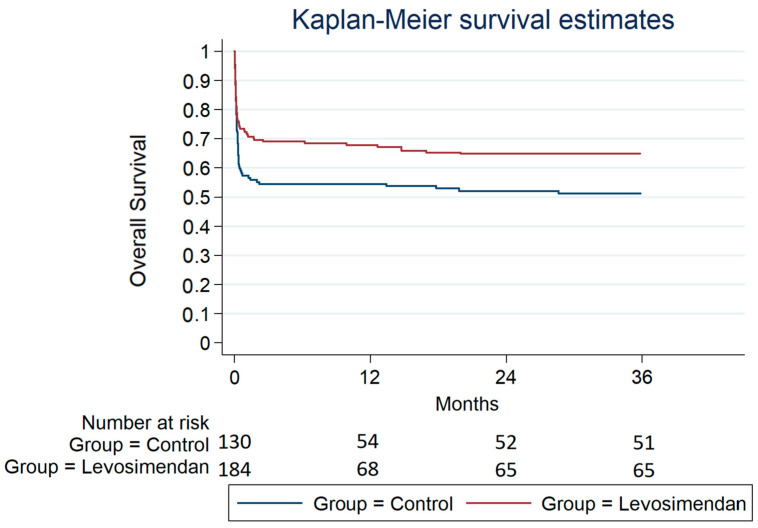
Overall survival from the date of operation within 3 years after surgery.

Figure 1, Figure 2 and Figure 3 depict the overall survival probability, estimated using the Kaplan–Meier (KM) method, within specific patient subgroups from the date of surgery. The KM plot revealed a significantly higher overall survival in the levosimendan group compared to the control group (*p* = 0.005, as determined by the log-rank test). Survival estimates at different timepoints for both subgroups consistently demonstrated a greater overall survival probability in the levosimendan group throughout the follow-up period. Notably, Figure 1 and Figure 2 display the survival probability of patient groups at 30 days and 1 year after the surgery date (*p* = 0.05 and *p* = 0.02 from the log-rank test, respectively). At 3 years post surgery, the control group exhibited a 51% survival rate, whereas the levosimendan group showed a higher rate of 65% (*p* = 0.005), as illustrated in Figure 3.

### 3.4. Postoperative Parameters

Table 4 displays the frequency and percentage of postoperative parameters within the two groups. A statistically significant difference was observed between the cohorts for all variables, with the exception of the duration of intra-aortic balloon pump (IABP) support, duration of support with inotropic drugs and catecholamines in the ICU, and hospitalization days.

**Figure 4:**
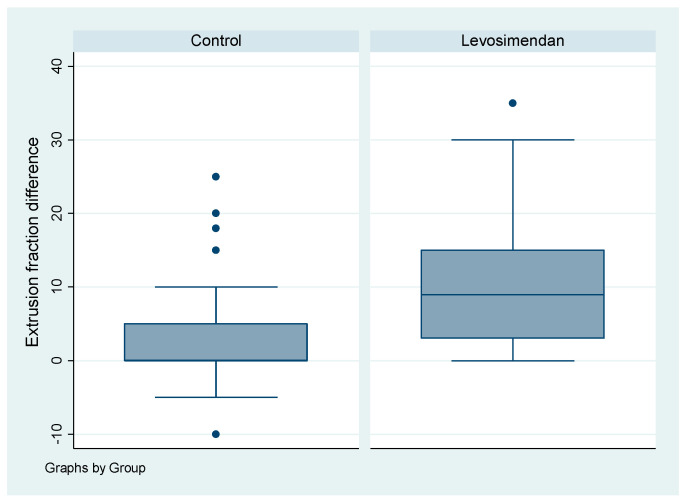
Boxplot of ejection fraction difference (ΔEF) by cohort. The dots represent the outliers, which are the points that stay out of the interval.

A statistically significant increase in the distribution of ejection fraction was observed in the group receiving the drug (*p* = 0.025). When comparing the difference in ejection fraction (ΔEF) between the two groups, a significantly higher disparity was noted in the levosimendan group (*p* < 0.001), as illustrated in Figure 4.

Patients in the levosimendan group exhibited easier weaning from the ventilator, with earlier extubation (*p* = 0.007) In terms of hospitalization and ICU stay, a tendency toward longer length was observed in the control group, although no statistical significance was found (*p* = 0.405 and *p* = 0.664, respectively). Similarly, levosimendan group demonstrated a tendency to lower use of catecholamines and inotropic support and mechanical circulatory support with IABP, but those differences were not statistically significant (*p* = 0.076 and *p* = 0.810, respectively). The control group had a significantly higher frequency of postoperative arrhythmias compared to the levosimendan group (53% vs. 40.76%, respectively, *p* = 0.031).

A similar pattern was observed regarding dyspnea and angina staging. It is noteworthy that only 4.62% of patients in the control group experienced Stage 0 angina postoperatively, which is considerably lower than the 35.87% observed in the levosimendan group.

**Figure 5:**
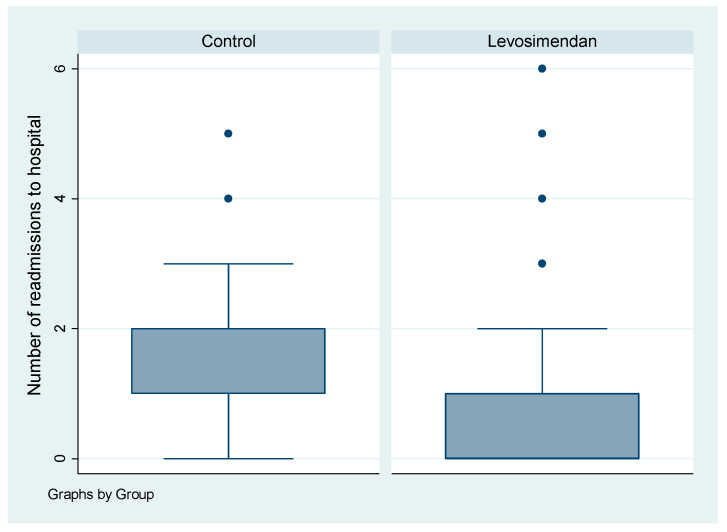
Boxplot of number of readmissions to hospital by cohort. The dots represent the outliers, which are the points that stay out of the interval.

A significantly greater proportion of patients in the control group had two or more hospital readmissions following surgery, in comparison to the levosimendan group (*p* < 0.001). These readmissions were primarily related to cardiovascular causes, particularly heart failure. Readmissions unrelated to heart failure were excluded from analysis (Figure 5).

### 3.5. Laboratory Hematological and Biochemical Parameters

The results of various blood parameters obtained at different timepoints from both patient cohorts are presented in Table 5.

The control group exhibited a significantly higher preoperative hemoglobin value (*p* = 0.003), indicating a greater tendency toward anemia in the levosimendan group. Hemoglobin levels were found to be higher in the levosimendan group patients on the day of surgery and the first postoperative day (*p* < 0.001 and *p* = 0.047, respectively). However, no significant difference was observed between the two groups in terms of hemoglobin values on the operation day, and on the second, third, and fourth postoperative days. The only significant difference in postoperative values was observed on the fifth postoperative day, favoring the control group.

**Figure 6:**
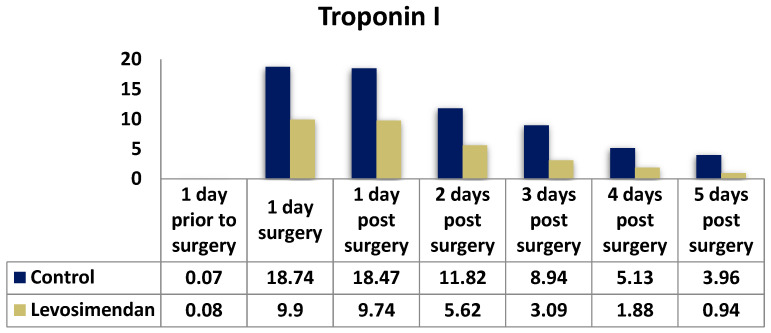
Distribution of troponin I levels in the two cohorts.

Troponin I levels (Figure 6) were consistently higher in the control group at all timepoints. Notably, the median troponin I level on the first postoperative day was 18.47 in the control group and 9.90 in the levosimendan group (*p* = 0.001).

**Figure 7:**
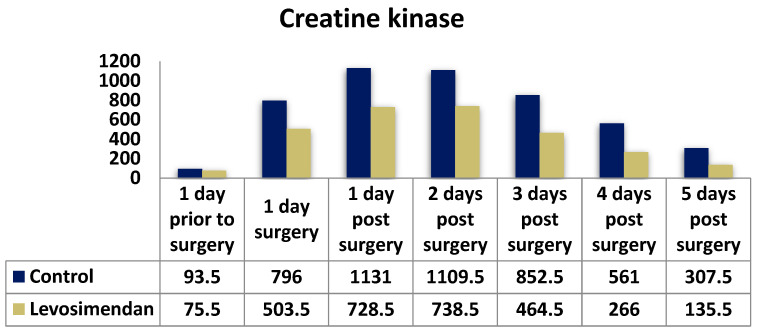
Distribution of CK levels in the two cohorts.

**Figure 8:**
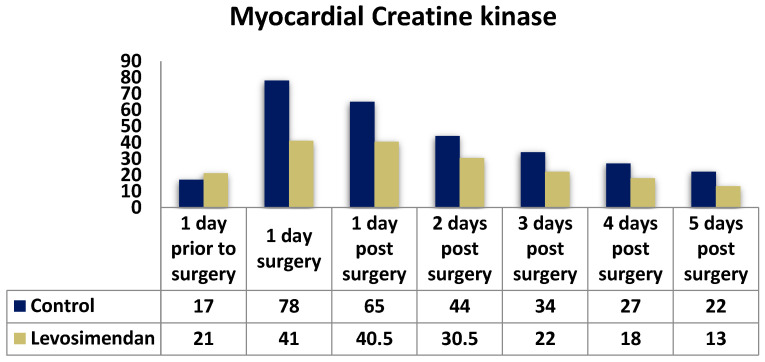
Distribution of CK-MB levels in the two cohorts.

Furthermore, a significant difference was observed with lower levels of CK (Figure 7) and CK-MB (Figure 8) in the levosimendan group across all timepoints, except for the day prior to surgery for CK.

## 4. Discussion

Survival was a major endpoint of this study. The primary finding of our study is that the perioperative administration of levosimendan improved short- and long-term survival following cardiac surgery. This finding aligns with the results of several meta-analyses. Landoni et al. demonstrated in their analysis that the levosimendan group had lower overall mortality rates (17.4% vs. 23.3%) [1]. Similarly, Maharaj et al. reported lower mortality rates in the levosimendan group (4.9% vs. 11.4%) [2], while Chen et al. found a mortality rate of 5.9% in the levosimendan group compared to 8.4% in the control group [4]. Belletti et al. conducted a large meta-analysis involving 28,280 patients from 177 trials and concluded that there was no difference in mortality between the groups receiving inotropes/vasopressors and the control group (31.7% vs. 31.8%) except for levosimendan, which showed improved survival [5]. Another study by Levin et al. demonstrated a significant improvement in 30 day survival with levosimendan compared to placebo in patients with preoperatively poor EF (3.8% vs. 12.4% mortality) [3]. Wang et al. also conducted a recent meta-analysis suggesting that levosimendan reduces death and adverse outcomes in patients with low EF undergoing cardiac surgery [6].

However, conflicting findings were reported by Chen et al., who found no statistically significant reduction in mortality favoring levosimendan in adult cardiac surgery patients (6.4% vs. 8.4%) [7]. Zhou et al. conducted a meta-analysis of 30 randomized controlled trials in 2018, showing a reduction in postoperative mortality with perioperative administration of levosimendan (5.8% vs. 8.5%) [8]. Qiang et al. included 25 studies with 3247 patients and found that levosimendan reduced mortality after cardiac surgery, particularly in patients with low LVEF [9]. Ng et al. published a meta-analysis in 2019 demonstrating a significant reduction in mortality with levosimendan compared to placebo, especially in subgroups with severe low LVEF (≤30%) and preoperative administration of levosimendan [10]. 

In their meta-analysis, Chen et al. conducted a comprehensive assessment encompassing 29 trials [11]. Their findings revealed a significant reduction in the risk of mortality associated with the use of levosimendan compared to placebo (odds ratio [OR]: 0.74; 95% confidence interval [CI]: 0.56–0.97). Similarly, in a recent retrospective analysis conducted by Liu et al., a survival benefit was demonstrated in the levosimendan group [12]. Caruba et al. conducted a study in 2022 involving a cohort of 1084 patients: 809 undergoing isolated coronary artery bypass grafting (CABG) and 275 undergoing combined surgery. They found no significant difference in mortality at day 90 between the levosimendan and placebo groups (*p* = 0.27) [13]. However, a significant interaction between the type of surgery and the study drug was observed (*p* = 0.004). Specifically, a decrease in mortality at day 90 was observed in the isolated CABG subgroup (*p* = 0.013), whereas no significant effect was observed in the combined surgery subgroup (*p* = 0.19). A meta-analysis involving six randomized controlled trials and a total of 1441 patients further confirmed the differential effect on mortality at day 30 between the two subgroups [13].

In light of these literature references, our findings are consistent with the overall trend suggesting improved survival with levosimendan use in cardiac surgery patients. However, it is important to note that there may be variations in the effect of levosimendan on mortality as a function of patient characteristics and study design. Further research and high-quality trials are needed to establish a more definitive understanding of the use of levosimendan in cardiac patients with preoperative low LVEF.

The evaluation of postoperative LVEF was a crucial and intriguing parameter in this study. As stated earlier, the patients included in this study exhibited severely decreased LVEF of <30% preoperatively. Substantial improvement in EF was observed in nearly all patients following surgery. Notably, a statistically significant higher distribution of EF was observed in the levosimendan group postoperatively (*p* = 0.025). Although the median EF was 30% in both groups, the distribution of values indicated better left-ventricular function in patients treated with levosimendan. This finding supports the hypothesis of the positive inotropic and cardioprotective effects of levosimendan. Furthermore, it is noteworthy that a significantly higher distribution of EF was observed in the control group preoperatively (*p* < 0.001). The median preoperative EF was 30% for the control group and 20% for the levosimendan group. Considering the higher degree of heart failure in the levosimendan group prior to the operation, the significant improvement in postoperative EF becomes even more meaningful. To strengthen our conclusion, we also conducted a statistical analysis comparing the preoperative and postoperative EF between the two groups. The levosimendan group exhibited a significantly higher difference in ejection fraction (ΔEF) compared to the control group (0 for the control group vs. 9 for the levosimendan group, *p* < 0.001). This parameter further reinforces the positive effect of levosimendan. These findings are consistent with numerous studies in the existing literature that also demonstrate an improvement in postoperative EF [12,13,14].

The administration of levosimendan exhibited a protective effect on lung function by reducing the duration of postoperative ventilation (*p* = 0.007), although there was no significant difference in ICU stay (*p* = 0.664) or in total hospital stay (*p* = 0.405) between the two groups. Our findings align with experimental evidence supporting the lung-protective properties of levosimendan [15,16]. Studies by Landoni et al. [1] and Lim et al. [17] demonstrated a noteworthy reduction in hospital stay among patients treated with levosimendan. Additionally, Treskatsch et al. [18] and Qiang et al. [9] reported that the levosimendan group experienced shorter durations of ICU stay and mechanical ventilation. However, Kodalli et al. [19], in their double-blind, randomized study involving 30 patients, failed to identify significant differences in ventilation duration and ICU stay. Overall, the accumulated evidence underscores the potential benefits of levosimendan in improving postoperative lung function and reducing ICU-related interventions, although further research is warranted to fully elucidate its impact in diverse clinical contexts.

The need for inotropic support and administration of catecholamines was slightly, yet not significantly lower in the levosimendan group patients (*p* = 0.076). If the higher cardiovascular risk of the treatment group of patients is taken into account, levosimendan shows a cardioprotective effect with a faster recovery of myocardium after treatment. These findings are generally supported by most literature reports with a reduced catecholamine requirement after preconditioning with levosimendan [20,21].

Postoperative arrhythmias, particularly postoperative atrial fibrillation (POAF), are common complications following cardiac surgery. The reported incidence of POAF in cardiothoracic surgery ranges from 16% to 46%, while, in non-cardiothoracic surgery, it varies between 0.4% and 12% [22,23]. POAF can occur throughout the postoperative period, with the highest occurrence observed between the second and fifth postoperative days. This complication is associated with increased morbidity, mortality, prolonged hospital stays, and higher healthcare costs. Numerous studies have aimed to identify predictors of POAF, with advanced age being the most consistent risk factor, followed by a history of atrial fibrillation, chronic obstructive pulmonary disease, and various operative characteristics [22]. Prophylactic administration of levosimendan has been shown to significantly reduce the incidence of POAF, particularly in patients undergoing isolated coronary artery bypass grafting (CABG) procedures, as reported by Abacilar et al. [23]. These findings are supported by De Hert et al., who conducted a randomized study involving three study groups and found that the group receiving levosimendan before surgery had significantly lower rates of postoperative atrial fibrillation compared to the groups receiving levosimendan intraoperatively or not receiving levosimendan but treated with milrinone instead [24]. In our study, postoperative arrhythmias were observed in a substantial number of patients in both groups. However, the frequency of such incidents was significantly higher in the control group (53%) compared to the levosimendan-treated group (40.76%) (*p* = 0.031). To comprehensively evaluate this finding, the preoperative prevalence of arrhythmias should also be considered. The comparison revealed a significant difference in the occurrence of arrhythmias before surgery between the two groups, with 19.23% in the control group and 30.43% in the levosimendan group (*p* = 0.030). This comparison demonstrates that, although arrhythmias were more prevalent in the levosimendan group, they experienced significantly fewer postoperative arrhythmias compared to the control group. These results collectively suggest a prophylactic antiarrhythmic effect of levosimendan.

CABG is a surgical procedure that often results in significant hemoglobin loss, necessitating blood-product transfusion in approximately 50% of patients [25,26]. Noteworthy differences were observed between the groups on the day of the operation, immediately after the patient’s transfer to the intensive care unit (ICU) (9.3 g/dL in the control group vs. 9.8 g/dL in the levosimendan group, *p* < 0.001), and on the first postoperative day (9.5 g/dL in the control group vs. 9.8 g/dL in the levosimendan group, *p* = 0.047), with the levosimendan group exhibiting superior hemoglobin levels. However, no significant differences were noted on the second, third, and fourth postoperative days (*p* = 0.956, 0.315, and 0.082, respectively). On the fifth postoperative day, a significant difference reemerged, albeit the control group exhibited higher levels this time (10.4 g/dL vs. 9.9 g/dL, *p* = 0.028). This discrepancy may be explained by the fact that the control patients had a higher median preoperative value; by the fifth postoperative day, both groups had reached levels similar to their baseline values, signifying recovery.

Every patient undergoing cardiac surgery experiences a certain degree of myocardial injury, which can lead to prolonged hospitalization and increased perioperative mortality rates [27]. Postoperative elevation of cardiac troponin (cTn) levels is observed in all cardiac surgery patients, even in the absence of postoperative myocardial infarction (MI), which can be attributed to various factors [28]. The type of surgery, choice of cardioplegic solution and its administration method, and intraoperative use of aprotinin all contribute to the release of cTn after surgery [27]. Studies have demonstrated the prognostic significance of postoperative troponin measurements in terms of short- and long-term outcomes [29,30]. However, it has been shown that coronary artery anastomosis alone during off-pump coronary artery bypass (OPCAB) surgery does not result in significant cTn release, unlike more complex procedures [31,32]. The frequent use of internal defibrillation before the end of cardiopulmonary bypass (CPB) does not contribute substantially to ischemic myocardial damage, as it only produces minor and transient elevations in cTn levels [33]. A meta-analysis by Zangrillo et al. revealed that levosimendan-treated patients exhibited lower cTn release peaks, suggesting a beneficial cardioprotective effect, which may translate into a reduced length of hospital stay [27].

To evaluate potential myocardial injury and monitor recovery, creatine kinase (CK), creatine kinase myocardial component (CKMB), and troponin I (cTnI) levels were measured daily. Both study groups exhibited similar trends, with a peak on the first postoperative day. However, cTnI levels significantly differed between the groups at all timepoints (*p* < 0.001). For instance, the median troponin I levels on the first postoperative day were 18.47 ng/mL in the control group and 9.90 ng/mL in the levosimendan group (*p* < 0.001). There were significant differences in the distribution of CKMB and CK between the two groups at all timepoints (*p* < 0.001). In summary, the control group demonstrated higher cardiac enzyme release, particularly within the initial 48 h, suggesting more pronounced myocardial damage.

The preservation of early cardiac function with levosimendan may contribute to improved global tissue perfusion and enhanced recovery from surgery. Patient quality of life is an equally significant aspect alongside survival rates. The levosimendan group demonstrated a notable inclination toward fewer postoperative symptoms. When comparing higher angina classes, a distinct advantage was observed in favor of the levosimendan group. Similarly, a similar pattern was noted for dyspnea. For instance, none of the control group patients reported NYHA class 0 (0%), whereas 10.33% of the levosimendan group patients indicated no physical activity limitations due to dyspnea following the operation. These findings support the conclusion that patients in the levosimendan group not only experienced significantly improved survival rates but also enjoyed a better quality of life with fewer postoperative symptoms.

The quality of life of patients who have survived cardiac surgery is equally important to their survival rates. In addition to assessing survival, we aimed to investigate an aspect that has not yet been addressed in the existing literature: the frequency of hospital readmissions due to heart failure-related issues. We recorded the number of new hospital readmissions within 3 years after surgery for all patients. This information was obtained by reviewing hospital archives and directly contacting the patients or their general practitioners during follow-up. Only readmissions related to cardiac causes were considered. A significantly higher proportion of patients in the control group experienced two or more readmissions after discharge, in comparison to the levosimendan group (*p* < 0.001). These findings provide further evidence of the long-term benefits of levosimendan in heart failure patients.

### Limitations

The present study had certain limitations, primarily due to its retrospective nature and the potential inequality between the studied groups. As mentioned earlier, there was significant heterogeneity in some of the preoperative parameters measured or recorded between the two groups. To minimize this, we consistently excluded patients with insufficient data documentation. However, this heterogeneity did not appear to significantly affect the conclusions drawn from the analysis of the results. Additionally, due to the retrospective design of our study, the standardization of catecholamine control was not implemented. It is challenging to determine the extent to which a standardized protocol for intravenous fluid volume and catecholamine dosage was followed in our patient cohort. In cases where patients underwent more complex interventions and initially had poorer ejection fractions, it is possible that a more conservative approach to fluid infusion volume was adopted, resulting in an increased need for vasopressors. These aspects could be clarified through a prospective investigation that incorporates comprehensive standardization of therapy control, thus allowing for a more accurate assessment of the true impact of levosimendan on the requirement for additional circulatory support. 

Despite the limitations of this study, it offers several advantages. The data collected accurately reflect the use of levosimendan in routine clinical practice. The inclusion of a substantial number of patients and the comprehensive recording of various parameters for each patient contribute to the robustness of this study and provide a representative addition to the existing literature on this topic. Furthermore, the long-term data on levosimendan utilization presented in this study, including follow-up periods of up to 5 years postoperatively, were not previously available in the literature.

## 5. Conclusions

In conclusion, this study provided evidence of the positive effects of administering levosimendan in cardiac surgery. It is important to note that levosimendan should be used as an additional treatment, complementing conventional medical and mechanical therapeutic approaches, which remain essential in the management of cardiothoracic surgical patients. To obtain a more accurate understanding of the precise benefits and potential drawbacks of levosimendan, further prospective randomized trials are warranted. These studies should focus on standardizing and comprehensively documenting perioperative therapy, allowing for a more precise quantification of the effects of levosimendan. By conducting such trials, we can enhance our knowledge and provide clearer guidelines for its clinical application. It is through robust and well-controlled research that we can truly elucidate the optimal role of levosimendan in cardiac surgery and improve patient outcomes.

## Figures and Tables

**Table 1:** Patients’ demographic characteristics at baseline.

Parameter	Control (N: 130)	Levosimendan (N: 184)	*p*-Value	OR (95% CI)
**Gender (N, %)**			0.551	0.748 (0.362–1.544)
Males	106 (81.54)	145 (78.80)		
Females	24 (18.46)	39 (21.20)		
**Age (years)**				
Median (IQR)	71 (63, 76)	69 (63, 76.50)	0.703	0.993 (0.969–1.018)
**Weight (kg)**				
Median (IQR)	82.15 (75, 88)	83.99, 85 (76, 90)	0.087	1.034 (0.968–1.104)
**Height (cm)**				
Median (IQR)	174.5 (168, 178)	175 (168, 178)	0.874	0.985 (0.934–1.038)
**Body mass index (BMI)**				
Median (IQR)	26.8 (25.40, 28.70)	27.6 (25.5, 30.38)	0.072	0.946 (0.785–1.139)
**Body surface area (BSA)**				
Median (IQR)	1.99 (1.85, 2.06)	2.01 (1.91, 2.10)	0.096	0.817 (0.461–1.451)

**Table 2:** Patients’ clinical characteristics prior to surgery.

Parameter	Control (N: 130)	Levosimendan (N: 184)	*p*-Value	OR (95% CI)
**Euroscore 1**				
Median (IQR)	15.72 (8.07, 27.90)	22.80 (11.59, 42.52)	**<0.001**	0.983 (0.951–1.016)
**Euroscore 2**				
Median (IQR)	5.43 (3.41, 10.46)	8.82 (5.09, 17.10)	**<0.001**	1.067 (0.995–1.145)
**ASA score (N, %)**			**0.048**	0.957 (0.544–1.681)
3	27 (20.00)	31 (16.85)		
4	89 (68.46)	116 (63.04)		
5	14 (10.77)	37 (20.11)		
**Type of surgery (N, %)**				
Elective/regular surgery	96 (73.85)	133 (72.28)	0.759	0.97 (0.425–2.215)
Emergency surgery	34 (26.15)	51 (27.72)		
**Simple/Combination surgery (N, %)**				
Simple surgery	95 (73.08)	118 (64.13)	0.095	0.934 (0.501–1.742)
Combination surgery	35 (26.92)	66 (35.87)		
**Acute myocardial infarction (AMI) (N, %)**				
No	94 (72.31)	137 (74.46)	0.671	0.806 (0.382–1.702)
Yes	36 (27.69)	47 (25.54)		
**Arterial hypertension (N, %)**				
No	9 (6.92)	8 (4.35)	0.321	1.33 (0.414–4.276)
Yes	121 (93.08)	176 (95.65)		
**Pulmonary hypertension (N, %)**				
No	119 (91.54)	128 (69.57)	**<0.001**	**3.711 (1.696–8.119)**
Yes	11 (8.46)	56 (30.43)		
**Hyperlipidemia (N, %)**				
No	35 (26.92)	37 (20.11)	0.157	1.34 (0.687–2.615)
Yes	95 (73.08)	147 (79.89)		
**Diabetes mellitus (N, %)**				
No	96 (73.85)	127 (69.02)	0.353	0.918 (0.491–1.717)
Yes	34 (26.15)	57 (30.98)		
**Arrhythmias (N, %)**				
No	105 (80.77)	128 (69.57)	**0.025**	1.137 (0.59–2.192)
Yes	25 (19.23)	56 (30.43)		
**Renal failure (N, %)**				
No	112 (86.15)	137 (74.46)	**0.012**	1.483 (0.71–3.098)
Yes	18 (13.85)	47 (25.54)		
**Chronic obstructive pulmonary disease (COPD) (N, %)**				
No	109 (83.85)	141 (76.63)	0.118	1.326 (0.659–2.667)
Yes	21 (16.15)	43 (23.37)		
**Extracardiac arteriopathy (N, %)**				
No	108 (83.08)	126 (68.48)	**0.003**	1.713 (0.862–3.404)
Yes	22 (16.92)	58 (31.52)		
**Cerebrovascular accidents (N, %)**				
No	124 (95.38)	159 (86.41)	**0.009**	**2.985 (1.069–8.337)**
Yes	6 (4.62)	25 (13.59)		
**NYHA classification (N, %)**			**0.032**	1.152 (0.671–1.976)
1	0 (0.00)	0 (0.00)		
2	7 (5.38)	8 (4.35)		
3	66 (50.76)	72 (31.13)		
4	57 (43.85)	104 (56.52)		
**CCS classification (N, %)**			0.124	0.939 (0.809–1.091)
0	3 (2.31)	3 (1.63)		
1	6 (4.62)	10 (5.43)		
2	18 (13.85)	24 (13.04)		
3	60 (46.15)	65 (33.00)		
4	43 (33.08)	82 (44.57)		
**Ejection fraction (EF)**				
Median (IQR)	30 (25, 30)	20 (18.5, 30)	**<0.001**	**0.886 (0.845–0.93)**

**Table 3:** Survival probability alongside 95% CIs at different timepoints from operation date by cohort.

Group	10 Days	30 Days	6 Months	1 Year	2 Years	3 Years
**Control** Median (IQR)	0.82 (0.75–0.88)	0.64 (0.55–0.71)	0.55 (0.46–0.63)	0.54 (0.45–0.62)	0.52 (0.43–0.60)	0.51 (0.42–0.59)
**Levosimendan** Median (IQR)	0.85 (0.79–0.90)	0.76 (0.69–0.82)	0.70 (0.62–0.76)	0.68 (0.61–0.75)	0.65 (0.58–0.72)	0.65 (0.57–0.71)

**Table 4:** Postoperative parameters in the two groups.

	Control (N: 130)	Levosimendan (N: 184)	*p*-Value
**Number of readmissions to hospital (N, %)**			**<0.001**
0	2 (2.74)	74 (54.41)	
1	25 (34.25)	36 (26.47)	
2	30 (41.10)	16 (11.76)	
3	8 (10.96)	5 (3.68)	
4	5 (6.85)	2 (1.47)	
5	3 (4.11)	1 (0.74)	
6	0 (0.00)	2 (1.47)	
Median (IQR)	2 (1, 2)	0 (0, 1)	
**Dyspnea staging (NYHA) (N, %)**			**<0.001**
0	0 (0)	19 (10.33)	
1	14 (10.77)	76 (41.30)	
2	48 (36.92)	34 (18.48)	
3	22 (16.92)	11 (5.98)	
4	46 (35.38)	44 (23.91)	
**Staging of angina (CCS) (N, %)**			**<0.001**
0	6 (4.62)	66 (35.87)	
1	45 (34.62)	67 (36.41)	
2	30 (23.08)	9 (4.89)	
3	19 (14.62)	7 (3.80)	
4	30 (23.08)	35 (19.02)	
**Postoperative EF**			
Median (IQR)	30.00 (25.00, 30.00)	30.00 (25.00, 39.50)	**0.025**
**Artificial ventilation (days)**			
Median (IQR)	2 (2, 4)	2 (1, 4)	**0.007**
**Support with IABP (days)**			
Median (IQR)	1 (0, 5.5)	2 (0, 4)	0.810
**Duration of support with inotropic drugs (days)**			
Median (IQR)	4 (3, 6)	4 (2, 6)	0.076
**Hospitalization days**			
Median (IQR)	15 (13, 21)	16 (12.50, 26.50)	0.405
**Postoperative arrhythmias (N, %)**			**0.031**
No	61 (46.92)	109 (59.24)	
Yes	69 (53.08)	75 (40.76)	
**Ejection fraction difference (ΔEF)**			
Median (IQR)	0 (0, 5)	9 (2.50, 9.50)	**<0.001**
**Intensive care unit (ICU) stay (days)**			
Median (IQR)	7 (4, 9)	6 (4, 10)	0.664

**Table 5:** Blood parameters at different timepoints in the two cohorts. The arithmetic median and interquartile range (IQR) values are indicated.

	Control (N: 130)	Levosimendan (N: 184)	*p*-Value
**Hemoglobin (g/dL); median (IQR)**			
Day prior to surgery	14.30 (12.50, 15.40)	13.40 (11.65, 14.95)	**0.003**
Day of surgery	9.30 (8.55, 10.00)	9.80 (9.90, 10.70)	**<0.001**
1st postop. day	9.50 (8.6, 10.50)	9.8 (8.90, 10.80)	**0.047**
2nd postop. day	9.60 (8.90, 10.40)	9.6 (8.90, 10.40)	0.956
3rd postop. day	9.70 (9, 10.40)	9.5 (8.80, 10.30)	0.315
4th postop. day	10 (9.20, 10.80)	9.70 (8.90,10.70)	0.082
5th postop. day	10.40 (9.40, 11.30)	9.90 (9.10, 10.90)	**0.028**
**Troponin I (TnI) (ng/mL); median (IQR)**			
Day prior to surgery	0.07 (0.03, 0.28)	0.08 (0.04, 0.52)	**0.028**
Day of surgery	18.74 (8.31, 50)	9.90 (4.05, 24.75)	**<0.001**
1st postop. day	18.47 (7.17, 50)	9.74 (4.88, 28.83)	**0.001**
2nd postop. day	11.82 (5.43, 38.14)	5.62 (2.26, 17.64)	**<0.001**
3rd postop. day	8.94 (3.56, 26.94)	3.09 (1.13, 10.68)	**<0.001**
4th postop. day	5.13 (2.12, 17.61)	1.88 (0.73, 6.01)	**<0.001**
5th postop. day	3.96 (1.32, 10.35)	0.94 (0.36, 3.51)	**<0.001**
**Creatine Kinase (CK) (U/L); median (IQR)**			
Day prior to surgery	93.50 (57, 147)	75.50 (48.50, 156)	0.114
Day of surgery	796 (453, 1530.50)	503.50 (288.25, 1010.25)	**<0.001**
1st postop. day	1131 (597, 2026)	728.50 (374.50, 1741.50)	**0.005**
2nd postop. day	1109.5 (565, 2725)	738.50 (309, 2116.50)	**0.014**
3rd postop. day	852.50 (387, 1990)	464.5 (166.5, 1314)	**<0.001**
4th postop. day	561 (193, 1293)	266 (99, 792.50)	**<0.001**
5th postop. day	307.50 (102, 884)	135.50 (61, 389.50)	**<0.001**
**Myocardial CK (CKMB) (U/L); median (IQR)**			
Day prior to surgery	17 (13,22)	21 (17,28)	**<0.001**
Day of surgery	78 (40.50, 202.50)	41 (27.25, 77.50)	**<0.001**
1st postop. day	65 (40,148)	40.5 (24.5, 74.5)	**<0.001**
2nd postop. day	44 (29, 90)	30.5 (19, 58)	**<0.001**
3rd postop. day	34 (21,71)	22 (14, 39)	**<0.001**
4th postop. day	27 (18,46)	18 (12, 28)	**<0.001**
5th postop. day	22 (16,36)	13 (9, 21)	**<0.001**

## Data Availability

The data presented in this study are available upon request from the corresponding author.
